# Physicochemical Properties and Consumer Acceptance of Hamburgers Processed with Chicken Meat Affected by Wooden Breast Myopathy

**DOI:** 10.3390/ani10122330

**Published:** 2020-12-07

**Authors:** Rodrigo Fortunato de Oliveira, Maísa Santos Fávero, Juliana Lolli Malagoli de Mello, Fábio Borba Ferrari, Erika Nayara Freire Cavalcanti, Rodrigo Alves de Souza, Mateus Roberto Pereira, Aline Giampietro-Ganeco, Erick Alonso Villegas-Cayllahua, Heloisa de Almeida Fidelis, Pedro Alves de Souza, Hirasilva Borba

**Affiliations:** 1 Department of Technology, Faculty of Agricultural and Veterinary Sciences, São Paulo State University, Via de Acesso Prof. Paulo Donato Castellane, s/n-Bairro Rural, Jaboticabal, São Paulo 14884-900, Brazil; maisa.sfavero@gmail.com (M.S.F.); julianalolli@zootecnista.com.br (J.L.M.d.M.); fbf_zoo@hotmail.com (F.B.F.); erikanayarac@gmail.com (E.N.F.C.); mateusscj2012@hotmail.com (M.R.P.); eav.cayllahua@unesp.br (E.A.V.-C.); heloisa.a.fidelis@gmail.com (H.d.A.F.); p.souza@unesp.br (P.A.d.S.); hirasilva.borba@unesp.br (H.B.); 2 Faculty of Animal Science and Food Engineering, Campus Fernando Costa, University of São Paulo, Avenida Duque de Caxias Norte, 225, Pirassununga, Sao Paulo 13635-900, Brazil; rodrigo.zootecnista@gmail.com (R.A.d.S.); giampietroganeco@gmail.com (A.G.-G.)

**Keywords:** chicken breast meat, frozen storage, meat quality, tenderness, wooden breast

## Abstract

**Simple Summary:**

The study of meat quality of modern birds and their respective myopathies is important to understand the influence of myopathy on the meat quality of these birds. The constant genetic evolution that birds have suffered and still suffer in the present is the most plausible cause of the onset of this myopathy. The processing of by-products, such as hamburgers, enters as an alternative to avoid losses that this myopathy generates in the poultry industry, with discards of chickens affected by the different degrees of myopathy in wooden breast.

**Abstract:**

Considering the increased incidence of wooden breast myopathy in broilers, the intake involves no threat to human health, indefinite etiology consumer rejection by appearance in such breasts, and the lack of studies on the industrial use of wooden breast. The objective of this study was evaluating the quality of hamburgers made with chicken meat affected by wooden breast. Breast samples from broilers slaughtered at 48-days-old were used. Normal (absence of myopathy), moderate degree (hardness only in one region of the breast) and severe degree (hardness over the entire length of the breast) samples were processed for the manufacture of hamburgers whose quality analyses (color, pH, cooking weight loss, shrinkage percentage, tenderness, storage weight loss, water activity, lipid oxidation, chemical composition and consumer test) were performed on non-stored samples (Day 0), and after storing at 30, 60, 90 and 120 days at −20 °C. There was a reduction (*p* < 0.05) in tenderness in samples of moderate-grade hamburgers (from 161.45 N to 289.40 N) after 120 days of storage. Hamburgers produced with chicken breast samples affected by wooden breast myopathy presented higher (*p* < 0.0001) fat concentration (5.32 g/100 g and 5.26 g/100 g, respectively, for the moderate and severe degree) than hamburgers made of normal samples (4.45 g/100 g). Lipid oxidation values increased, which exceeded the limit of rancidity detection, independent of myopathy. The consumers equally appreciated the aroma, flavor, and texture, and rated their overall acceptance as similar regardless of the quality of chicken meat. Chicken breast hamburgers with wooden breast myopathy is a viable alternative for the poultry industry.

## 1. Introduction

The world’s chicken meat production has grown markedly in the last few decades, with a 3% increase estimated for 2020 in relation to the 95.5 million tons produced in the year 2019 [1]. Today’s successful production and exports of chicken meat are a result of measures adopted by the poultry industry, with high growth rates, minor feed conversion ratios and elevated meat production [2] that meet the market demands. However, problems that compromise the functionality and quality of chicken meat have accompanied genetic selections [3], and one of the serious problems affecting chicken meat quality is wooden breast myopathy [4]. The underlying causes of this abnormality have not been fully unveiled, but it is known that there is a connection with fast growth and large chicken breasts [5].

The wooden breast abnormality is characterized by pale breasts and breasts with hardened areas, exudation, and bleeding, and sometimes, wooden breast may be associated with other myopathies, such as white striping along the muscle surface [5,6]. Wooden breast myopathy may be found at different degrees of severity [4]. Despite the remarkable macroscopic changes in wooden breast chicken, the intake of wooden breast meat involves no threat to human health [7]. The wooden breast myopathy is not yet covered by legislation in several countries, which has caused economic problems for the sector [8].

The myopathy can only be detected manually, during production [6]. This myopathy provides a hardened meat with color unappealing to the consumer, which no longer has the sensory standards for direct sale and is used for the preparation of industrialized meat products, such as hamburgers [9], animal feed or is discarded. The hamburger is an option for the technological use of chicken breast affected by wooden breast myopathy, considering its commercial value. However, Brazilian law provides for the use of breasts affected by wooden breast myopathy in the production of industrialized meat products [10]. Nevertheless, the lack of research involving the storage of hamburgers prepared with breast affected by the myopathy is highlighted. In this context, studies involving the effect of time and storage conditions of meat products are essential factors in meat processing, since problems related to storage stability are common [11].

Studies have reported alternatives for the use of wooden breasts in the preparation of meat products, such as nuggets, sausage, [12,13] and chicken emulsion-type hamburgers [9]. In a recent study, observed that wooden breast can be used in the formulation of chicken sausage either combined or not combined with normal breast [14]. However, there is a gap in knowledge regarding the effect of frozen storage conditions on the quality of emulsion-type wooden breast chicken hamburger. Given the above, this study aimed to evaluate the effect of a long-term frozen storage on the physicochemical quality of hamburgers prepared with chicken breast fillets affected by moderate and severe degree of Wooden Breast myopathy.

## 2. Materials and Methods

This study was conducted in the Laboratory of Analysis of Animal Origin Foods of the Faculty of Agrarian and Veterinary Sciences of UNESP, Campus Jaboticabal, São Paulo, Brazil (21°08′ S, 48°11′ W, 583 m altitude).

### 2.1. Sample Collection and Experimental Procedure

**Figure 1:**
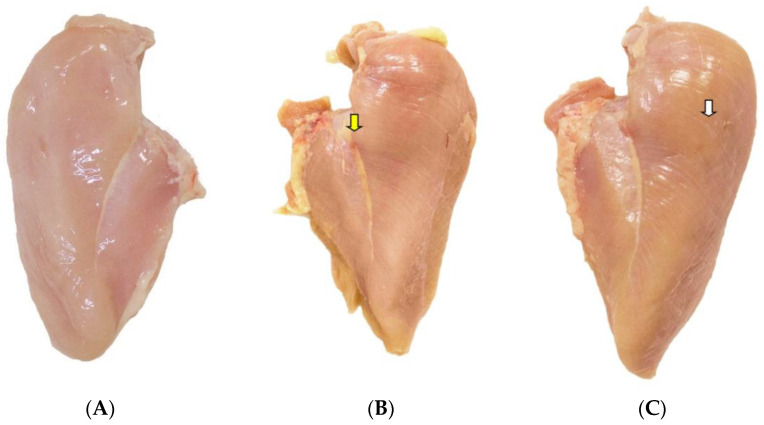
Normal pectoralis major muscle (**A**), and pectoralis major muscle affected by the moderate (**B**) and severe (**C**) degree of wooden breast in Ross chickens (AP 95). There are whitish striations in the upper quadrant of the muscle part (white arrow) as well as reddish (petechiae) and hardened areas in the middle and lower portions of the muscle (yellow arrows).

Muscle samples of Pectoralis Major affected by “Wooden Breast” myopathy were collected during one sampling session from male Ross chickens (AP 95) in a commercial slaughterhouse (SP, Nuporanga, Brazil) inspected by the Federal Inspection Service. The samples were classified according to [2,6] and according to the severity degree of the myopathy (moderate hardness found only in the cranial region or caudal region of the breast fillet; severe hardness verified over the entire length of the breast fillet) and control samples (samples without myopathies) were also collected (±10 kg per group—Figure 1). The chickens were reared in a traditional intensive system and slaughtered at 48 days old. After boning and characterization (one-hour postmortem) of the myopathy’s severity degree, the samples were separated within each anomaly category and transported to the university laboratory under refrigerated conditions (±4 °C).

Four hours after slaughter (establishment of rigor mortis), the collected samples from each evaluated group, after discarding connective tissue, were cut into cubes and ground in an Eberle manual meat grinder with a 0.005 m disc. In the hamburger formulation (Table 1), chicken skin was added as a percentage of fat before grinding. After grinding, the ingredients were added, and the homogenized mixture obtained was pressed and molded with a 0.125 m diameter manual hamburger, obtaining hamburgers with a net weight of 0.1 kg each.

After processing, the hamburgers were wrapped in polyethylene bags, packed in cardboard cartridges and stored in a freezer at −20 °C. Physicochemical analyses described below were performed on non-stored samples (Day 0) and samples stored at 30, 60, 90 and 120 days. In each period, 60 hamburgers were used, 20 of each severity degree of the myopathy (control, moderate and severe), totaling at the end of 120 days of storage, 300 hamburgers used, 100 of each severity degree of the myopathy.

### 2.2. Methods

#### 2.2.1. Hamburger Color

The color was determined using the Minolta Chrome Meter CR-400 (Konica Minolta Sensing, Inc., Osaka, Japan) colorimeter (settings: diffused lighting/0 viewing angle, illuminant D65, specular component included) calibrated to a white standard, using the CIELAB system (L*, a* and b*). Parameters, such as lightness (L*), redness (a*) and yellowness (b*), were evaluated at three different positions on the surface of raw and thawed chicken hamburger.

#### 2.2.2. pH, Cooking Weight Loss, Shrinkage Percentage and Tenderness

The pH was determined in triplicate using a Testo digital pH-meter (Testo 205, Testo Inc., Sparta, NJ, USA) fitted with a penetration electrode by direct insertion into the raw and thawed hamburger.

The weight of hamburgers before being frozen was determined on an analytical scale. Subsequently, the products were subjected to the cooking process on an preheated electric grill (George Foreman GBZ80, Lake City, FL, USA) according to Mello et al. [15] still frozen for 10 min (five minutes on each side), enough time for the sample to reach the internal temperature of 76 °C, according to the electric grill manual. Samples were cooled at room temperature, the excess fat from the hamburgers was removed with a paper towel so that its mass was again determined for evaluation of cooking weight loss (CWL), whose results were obtained by the difference between the initial and final weights and expressed as percentage. To determine the shrinkage or retraction percentage (SP), the still frozen hamburger had its average diameter determined by measuring the cross section in three distinct regions, using a 6′′ digital pachymeter (Zaas Precision, AMATOOLS Commercial e Importadora Ltd., Piracicaba, São Paulo, Brazil). After the cooking process, the diameter was determined again. From each cooked sample, three square subsamples, with known area (0.0625 m) were cut for the hardness analysis on a texturometer (TAXT2i, Stable Micro Systems, LTD., Godalming, UK), fitted with a compression platen (*p*/75, 0.075-m aluminium platen (TAXT2i, Stable Micro Systems, LTD., Godalming, UK) using the following settings: pretest speed 0.002 m·s^−1^; posttest speed 0.005 m·s^−1^; strain 50%; time 5.0 s; trigger type, auto; trigger force 0.005 kg. The results were expressed in Newtons (N).

#### 2.2.3. Storage Weight Loss, Water Activity and Lipid Oxidation

Raw and thawed chicken hamburger sub-samples were inserted into the container (with a capacity of 0.015 l) of Aqualab (Decal Devices Inc., Brooklyn, NY, USA) water activity analyzer (WA), which uses the dew point principle [16].

Storage weight loss was defined as the difference between the initial and final weight of each thawed sample before and after storage, expressed as a percentage.

Lipid oxidation was determined in all raw and thawed samples by the test of thiobarbituric acid reactive substances (TBARs), according to the methodology described by Vyncke [17], which uses trichloroacetic acid for the extraction of 0.005 kg of ground sample. After reaction under heating with thiobarbituric acid, a reading at the 538 nm wavelength is performed and the result is expressed in malonaldehyde mg (MDA)/kg sample.

#### 2.2.4. Chemical Composition

The chemical composition was determined in all samples by analysis of moisture (950.46—Moisture in Meat), protein (977.14—Nitrogen in Meat) and ashes (920.153—Ash of Meat) according to the procedures recommended by the Association of Official Analytical Chemists [16] and fat was determined in all samples by the method described by Bligh and Dyer [18].

#### 2.2.5. Consumer Test

An acceptance test with hedonic scale was used for the sensorial evaluation, using a scale of 9 points (9 = extremely like, 5 = neither like nor dislike, and 1 = extremely dislike), commonly used for meat evaluation [19]. The hamburger were rated for sensory attributes by 120 untrained participants (80 women and 40 men, aged between 18 and 45 years), who were recruited among students and staff members of UNESP, Jaboticabal Campus. Briefly, 1 cm-thick slices were cut, starting from the middle of the hamburger and cooked in an electric grill (George Foreman GBZ80) for 10 min (five minutes on each side), enough time for the sample to reach the internal temperature of 76 °C, according to the electric grill manual. One slice of cooked hamburger of each severity degree of the myopathy were coded with three-digit numbers and their order was randomized for each assessor. Water and unsalted crackers were provided for cleansing the palate between evaluations. Participants were given an evaluation form with the nine-point hedonic scale to assess aroma, flavor, texture, appearance and overall acceptance. The evaluations were carried out in individual booths. A consumer test was conducted after verifying the compliance of the microbiological parameters established by the Brazilian legislation for total and thermotolerant coliforms at 45 °C/kg, *Salmonella* spp./0.025 kg, coagulase positive Staphylococci/g, mesophilic bacteria/kg and psychotrophic bacteria/kg [20]. The study followed the ethical requirements of the sensory laboratory approved by the Paulista State University research ethics committee on 23 September 2020 (Ethic code: 4.294.461/CAAE 33846220.6.0000.9029), and informed consent was signed by the panelists.

### 2.3. Statistical Analysis

The data obtained from the physicochemical analyses were analyzed through a completely randomized experimental design (CRD) in a 3 × 5 factorial scheme (three myopathy severity degrees and five storage periods) with 20 replications. The data obtained in the chemical composition and consumer test were analyzed through a completely randomized experimental design (CRD) with 20 and 120 replications, respectively. Results were analyzed through the General Linear Models procedure of the Statistical Analysis System (SAS Institute Inc., 2002–2003, Cary, NC, USA). All data were tested by analysis of variance (ANOVA) and compared by Tukey test at a significance level of 5%.

## 3. Results

### 3.1. Hamburger Color

There was a significant interaction between severity degree and storage period for lightness (L*), redness (a*) and yellowness (b*) in chicken hamburger samples (Table 2). Samples of hamburger produced with chicken meat affected by wooden breast myopathy and stored (with the exception of 60 days storage) or not, showed an increase (*p* < 0.0001) in L* value compared to normal samples. During storage, both hamburger samples made with normal breasts and breasts with myopathy showed an increase (*p* < 0.0001) in L* value, with the maximum brightness at 60 days. Fresh hamburger samples stored for 30 days showed an increase (*p* < 0.0001) in a* value in relation to the severity degree. During storage, both hamburger samples made with normal breasts and breasts affected by moderate myopathy degree showed a reduction (*p* < 0.0001) in a* value, different from the severe degree that increased at 120 days of storage.

Regarding the yellowness, hamburger samples not stored and stored for 30 days and produced with breast affected by severe degree of myopathy presented higher b* value (9.95 and 12.96, respectively) than samples produced with normal breast (7.92 and 10.37, respectively) or those produced with the breast affected by moderate degree (9.07 and 11.50, respectively) of wooden breast myopathy. After 60 days of storage, there was a variation in the b* value among the samples affected by the different severity degrees of myopathy, in which hamburger samples produced with normal breasts presented higher b* value compared to hamburgers produced with breast affected by wooden breast myopathy. Considering each severity degree during the storage process, hamburger samples produced with normal breast increased yellowness from 7.92 to 13.67; hamburgers samples produced with breast affected by moderate degree of wooden breast myopathy increased from 9.07 to 14.30 and hamburger samples produced by breast affected by the severe degree of myopathy increased from 9.95 to 13.44.

### 3.2. pH, Cooking Weight Loss, Shrinkage Percentage and Tenderness

There was a significant interaction between severity degree and storage period for the variables pH, cooking weight loss (CWL) and shrinkage percentage (SP) in chicken hamburger samples (Table 3). Hamburgers produced with chicken meat affected by the myopathy had higher (*p* <0.0001) pH than hamburgers produced with normal chicken meat. There was an effect (*p* < 0.0001) of storage on the pH of hamburgers produced with breast meat, regardless of the myopathy, which reduced the pH value up to 120 days of storage.

Hamburger samples from chicken affected by wooden breast myopathy showed higher cooking weight loss and shrinkage percentage up to 60 days of storage than hamburgers produced with normal chicken meat. Increased cooking weight loss was observed up to 60 days and, after, reduced CWL up to 120 days in all samples. The shrinkage percentage increased up to 90 days of storage for hamburgers produced with normal breasts and up to 60 days breasts of storage for hamburgers produced with a moderate and severe degree of wooden breast myopathy. Afterwards, the shrinkage percentage decreased at 120 days, for all samples studied.

There was interaction (*p* < 0.0001) between factors for tenderness in chicken hamburgers. Hamburgers from breast samples affected by severe degree of myopathy were softer after 60 days of storage when compared to hamburgers produced with normal and moderate degree of wooden breast myopathy. During the storage process, a reduction in tenderness was observed in all hamburger samples.

### 3.3. Storage Weight Loss, Water Activity and Lipid Oxidation

There was a significant interaction between severity degree of wooden breast myopathy and storage period for storage weight loss (SWL), water activity (WA) and lipid oxidation (TBARS) (Table 4).

The hamburgers produced with meat affected by the severe degree of wooden breast myopathy showed greater (*p* < 0.0001) storage weight loss (Table 4) at 30, 90 and 120 days, due to the lower water retention capacity presented in the raw material used for the hamburger production. Weight loss in hamburger storage increased from 1.56% (normal samples) to 12.76% (affected by the severe degree of the myopathy).

Chicken hamburgers produced with meat affected by severe degree of wooden breast myopathy showed less variation in lipid oxidation values after 120 days of freezing compared to normal chicken samples. Hamburgers produced with chicken breast affected by severe degree of myopathy ranged from 1.69 to 4.09 mg MDA/kg, whereas in hamburgers produced with normal samples, the oxidation ranged from 2.32 to 9.31 mg MDA/kg within 120 days of freezing.

### 3.4. Chemical Composition

The use of meat with different severity degrees of wooden breast myopathy had no effect (*p* = 0.5441) on the mineral matter concentration of chicken hamburgers.

The moisture of hamburgers did not show significant variation (*p* = 0.8786) between the severity degrees (Table 5).

Hamburgers produced with samples of chickens with moderate and severe chicken breast myopathy showed higher lipid concentrations than hamburgers produced with normal chicken samples. The use of meat with different severity degrees of wooden breast myopathy had no effect (*p* = 0.0620) on the protein concentration of chicken hamburgers.

### 3.5. Consumer Test

Among the five evaluated attributes, only “appearance” showed a significant difference (*p* = 0.01) between the three formulations (Table 6), being that the hamburger made with chicken breast affected by the severe degree of myopathy showed better appearance than hamburgers made with normal chicken breast and chicken breast with moderate degree of myopathy.

## 4. Discussion

The higher values of lightness and yellowness found in hamburgers produced with breast affected by wooden breast myopathy may be explained, respectively, due to the used raw material presenting changes in muscle tissue after histological degeneration in the affected muscles and due to the characteristic increase in the amount of fat in the fillets used to produce hamburgers [12,21]. The prolonged frozen storage can promote oxidative processes and with this can influence the product color (mostly lightness and yellowness). The oxidation process itself probably caused a decrease in the value of a* in those produced with normal chicken breast and in those affected by moderate degree of wooden breast myopathy when compared to hamburgers produced with chicken meat with a severe degree of myopathy. The reduction in a* value after 120 days of storage probably occurred due to the interaction of pigments with lipid oxidation products [22], promoting the denaturation of myoglobin.

These high pH values in hamburgers produced with chicken meat affected by wooden breast myopathy indicate that there was an influence of the pH value of raw meat, because the low glycolysis presented after slaughter in chicken breast affected by this myopathy results in a higher pH. In detail, the higher final pH found in fillets with wooden breast myopathy is probably due to the decreased glycolytic potential, energy status and changes in metabolic pathways [23,24].

Cooking weight loss is considered to be one of the most important functional properties of meat products [25]. Qin [13] reported that there are no differences in the cooking loss of ground beef nuggets and sausages made up of up to 100% wooden breast meat and normal meat, independent of the grinding of the meat (pore size). Similar results were found by Kozačins et al. [11], who evaluated hamburgers produced with chicken meat affected by wooden breast myopathy and found no influence on the variable cooking weight loss. The authors also suggest that the grinding of fillets affected by wooden breast myopathy may reduce the negative influence of the myopathy condition on the cooking weight loss of breast meat. It is reported that, after storage, the cooking weight loss of meat affected by wooden breast myopathy is higher than normal breast [2] due to the lower water retention capacity. These results indicate that the wooden breast myopathy has a negative effect, increasing the losses (CWL and SP) up to 60 days of storage.

Among all parameters, tenderness stands out with great importance for the consumer [26]. The greatest tenderness observed in hamburgers made from breast meat with severe degree of myopathy in this research may have been caused by fiber degeneration and reduced salt-soluble myofibrillar protein content [12].

Hamburgers produced from breast meat, regardless of the myopathy, did not preserve the water activity (WA) values during the 120 days of freezing storage. Storage under freezing is the indicated method for preserving the quality of products considered perishable, as these foods have higher values for water activity and, when subjected to the freezing process, show a decrease in these values. In chicken hamburgers, regardless of the occurrence or absence of myopathy, lower values of water activity were observed in products that were frozen, in different periods of analysis, reducing the chances of suffering microbial action.

The MDA values presented appear high already at day 0 (on average, 1.95 mg MDA/kg sample). When compared to literature, these results do not corroborate with other similar studies reported values around 0.030 mg MDA/kg sample [27,28].

Campo et al. [29] have shown that TBARS values should be between 2 and 3 MDA/kg of product, so that the flavor and rancidity odor are noticeable to the consumer. The increase in TBARS values in chicken meat products results from their higher susceptibility to oxidation, as it contains a higher amount of polyunsaturated fatty acids. In hamburgers, TBARS values increased significantly at 120 days of storage in all treatments, probably due to the oxidation of polyunsaturated fatty acids [30].

Assuming that 3 mg MDA/kg sample is the rancidity perception threshold, values at days 0, 30 and 60 are around 2.0 mg MDA/kg sample; therefore, it is suggested that a 60-day freezing storage for hamburgers made with normal breast and breasts with moderate and severe degrees of wooden breast myopathy, with no rancidity observed. Soglia et al. [7] reported higher TBARS values in breasts affected by wooden breast myopathy, unlike what was observed in hamburgers formulated with breasts affected by wooden breasts in the present study. This behavior is justified by the fact that hamburgers are heterogeneous products, that are processed and ground, thus diluting the effect of the wooden breast anomaly on the lipid oxidation process. The TBARS results of this study may also be explained by the water activity of the products (Table 4), being that the water activity decrease promotes a higher lipid oxidation in the products.

The higher lipid oxidation (Table 4) observed in chicken hamburgers after 120 days of storage could be a result of higher lipid concentration, and despite the increased fat concentration in hamburgers after 120 days of storage (Table 5), hamburgers produced with meat affected by wooden breast suffered less lipid oxidation compared to normal samples. The increases in hamburger TBARS values probably resulted from changes in protein membrane structure and the release of iron from the carrier protein, which reacts with oxygen, thus accelerating the oxidation rate [31].

Variations in the percentage of fat in hamburgers may be related to the lack of uniformity, although the wooden breast was expected to present a higher fat content due to the lipidosis process. Studies have shown that the occurrence of wooden breast negatively affects some aspects of meat, including its histology, nutritional and chemical composition, and technological parameters [5]. The myodegenerative processes in wooden breast muscles cause alterations in the water-holding capacity and induce higher levels of lipid [32], which directly affects the processing of chicken meat [2,6,12,32].

The results of the consumer test showed the potential use of chicken breast affected by wooden breast myopathy in the making of hamburgers, since the participants equally appreciated the aroma, flavor, and texture, and rated their overall acceptance as similar regardless of the quality of chicken meat.

## 5. Conclusions

Hamburgers made with chicken meat affected by wooden breast myopathy showed the same reduction in water activity with storage and the same protein concentration as hamburgers produced with normal chicken meat. The raw material used in hamburgers produced with meat affected by wooden breast affected the physical quality of the samples, causing greater lightness, redness, yellowness, pH and CWL. Hamburgers produced with chicken breast affected by wooden breast showed regular oxidative stability. Hamburgers did not show any noticeable differences for consumers, as they were not able to detect the differences in the sensory characteristics of these products made with normal chicken breast and with the moderate and severe degrees of wooden breast myopathy.

## Figures and Tables

**Table 1:** Formulation used in the elaboration of hamburgers produced with chicken breast without myopathies and affected by wooden breast myopathy.

Ingredients	g/100 g
Breast filet	81.55
Chicken skin	10.0
Textured soy protein	3.55
Water (ice)	2.0
Iodised salt	1.5
Garlic paste	0.30
Ground white pepper	0.10
Citric acid (antioxidant)	1.0
Total	100

**Table 2:** Lightness (L*), redness (a*) and yellowness (b*) mean values of hamburgers produced with chicken breast of Ross broiler chickens (AP95) without myopathies and affected by wooden breast myopathy after different storage periods.

L*					
A (*n* = 20) ^1^	SD (*n* = 20) ^2^			*p*-value	
	Normal	Moderate	Severe		
Start	52.87 ± 0.49 ^Ec^	57.70 ± 0.51 ^Cb^	60.03 ± 0.49 ^Ca^		
30 days	58.61 ± 0.49 ^Cb^	62.86 ± 0.49 ^Aa^	64.11 ± 0.64 ^Aa^	(SD)	<0.000
60 days	64.91 ± 0.49 ^Aa^	63.65 ± 0.49 ^Aab^	62.63 ± 0.49 ^ABb^	(A)	<0.0001
90 days	60.01 ± 0.49 ^Bb^	58.78 ± 0.46 ^Cb^	62.12 ± 0.52 ^Ba^	(SD × A)	<0.0001
120 days	57.13 ± 0.49 ^Dc^	60.47 ± 0.44 ^Ba^	59.13 ± 0.40 ^Cb^		
a*					
A (*n* = 20) ^1^	Normal	Moderate	Severe	*p*-value	
Start	0.34 ± 0.10 ^ABb^	1.40 ± 0.10 ^Aa^	1.35 ± 0.10 ^Ba^		
30 days	0.55 ± 0.10 ^Ab^	1.21 ± 0.11 ^Aa^	1.35 ± 0.13 ^Ba^	(SD)	<0.0001
60 days	0.18 ± 0.10 ^Ba^	−0.16 ± 0.10 ^Bb^	0.23 ± 0.10 ^Ba^	(A)	<0.0001
90 days	−0.26 ± 0.10 ^Cb^	0.03 ± 0.10 ^Ba^	−1.35 ± 0.11 ^Dc^	(SD × A)	<0.0001
120 days	−0.76 ± 0.11 ^Db^	−1.15 ± 0.12 ^Cc^	2.15 ± 0.10 ^Aa^		
b*					
A (*n* = 20) ^1^	Normal	Moderate	Severe	*p*-value	
Start	7.92 ± 0.27 ^Dc^	9.07 ± 0.27 ^Cb^	9.95 ± 0.27 ^Ca^		
30 days	10.37 ± 0.27 ^Bc^	11.50 ± 0.27 ^Bb^	12.96 ± 0.35 ^ABa^	(SD)	<0.0001
60 days	13.28 ± 0.27 ^Aa^	12.02 ± 0.27 ^Bb^	12.22 ± 0.27 ^Bb^	(A)	<0.0001
90 days	9.32 ± 0.27 ^Ca^	8.62 ± 0.25 ^Ca^	8.92 ± 0.28 ^Da^	(SD × A)	<0.0001
120 days	13.67 ± 0.27 ^Aab^	14.30 ± 0.24 ^Aa^	13.44 ± 0.22 ^Ab^		

^1/A–E^ Means followed by different capital letters in the same column differ from each other by the Tukey test (*p* < 0.05). ^2/a–c^ Means followed by different lowercase letters on the same line differ from each other by the Tukey test (*p* < 0.05). A—Storage, SD—Severity degree.

**Table 3:** Mean values of pH, cooking weight loss (CWL), shrinkage percentage (SP) and hardness of hamburgers produced from Ross broiler chicken (AP95) without myopathies and affected by wooden breast myopathy after different storage periods.

**pH**					
	SD (*n* = 20) ^2^			*p*-value	
A (*n* = 20) ^1^	Normal	Moderate	Severe		
Start	5.847 ± 0.005 ^Ac^	5.972 ± 0.005 ^Ab^	5.997 ± 0.005 ^Aa^		
30 days	5.656 ± 0.005 ^Db^	5.689 ± 0.005 ^Ea^	5.656 ± 0.007 ^Eb^	(SD)	<0.0001
60 days	5.716 ± 0.005 ^Cb^	5.807 ± 0.005 ^Ca^	5.795 ± 0.005 ^Ca^	(A)	<0.0001
90 days	5.714 ± 0.005 ^Cc^	5.791 ± 0.005 ^Da^	5.775 ± 0.005 ^Db^	(SD × A)	<0.0001
120 days	5.783 ± 0.006 ^Bb^	5.879 ± 0.006 ^Ba^	5.882 ± 0.005 ^Ba^		
**CWL (%)**					
A (*n* = 20) ^1^	Normal	Moderate	Severe	*p*-value	
Start	28.51 ± 0.64 ^Dc^	32.58 ± 0.57 ^Cb^	38.44 ± 0.57 ^Ca^		
30 days	32.86 ± 0.62 ^Cc^	38.61 ± 0.57 ^ABb^	40.16 ± 0.57 ^Ba^	(SD)	<0.0001
60 days	36.73 ± 0.57 ^Bc^	39.62 ± 0.57 ^Ab^	42.12 ± 0.57 ^Aa^	(A)	<0.0001
90 days	38.39 ± 0.57 ^Aa^	37.81 ± 0.57 ^Ba^	37.55 ± 0.57 ^Ca^	(SD × A)	<0.0001
120 days	17.31 ± 0.51 ^Da^	16.45 ± 0.46 ^Ea^	16.08 ± 0.45 ^Ca^		
**SP (%)**					
A (*n* = 20) ^1^	Normal	Moderate	Severe	*p*-value	
Start	21.41 ± 0.49 ^Cc^	24.13 ± 0.44 ^Cb^	25.71 ± 0.44 ^Aa^		
30 days	22.28 ± 0.44 ^BCb^	25.61 ± 0.44 ^Ba^	25.71 ± 0.44 ^Aa^	(SD)	<0.0001
60 days	22.97 ± 0.44 ^ABb^	26.89 ± 0.44 ^Aa^	26.50 ± 0.44 ^Aa^	(A)	<0.0001
90 days	23.86 ± 0.45 ^Aa^	22.65 ± 0.44 ^Da^	23.12 ± 0.44 ^Ba^	(SD × A)	<0.0001
120 days	17.31 ± 0.51 ^Da^	16.45 ± 0.46 ^Ea^	16.08 ± 0.45 ^Ca^		
**Hardness (N)**					
A (*n* = 20) ^1^	Normal	Moderate	Severe	*p*-value	
Start	167.74 ± 6.75 ^Da^	161.45 ± 6.75 ^Da^	154.75 ± 6.75 ^Ca^		
30 days	174.70 ± 6.92 ^Da^	190.05 ± 6.75 ^Ca^	188.00 ± 6.75 ^BCa^	(SD)	<0.0001
60 days	226.06 ± 6.75 ^Ca^	230.37 ± 6.75 ^Ba^	198.81 ± 6.75 ^ABb^	(A)	<0.0001
90 days	283.50 ± 6.92 ^Ba^	235.94 ± 6.75 ^Bb^	214.92 ± 6.75 ^Ac^	(SD × A)	<0.0001
120 days	401.50 ± 8.37 ^Aa^	289.40 ± 7.32 ^Ab^	171.82 ± 6.92 ^Cc^		

^1 A–E^ Means followed by different capital letters in the same column differ from each other by the Tukey test (*p* < 0.05). ^2/a–c^ Means followed by different lowercase letters on the same line differ from each other by the Tukey test (*p* < 0.05). A—Storage, SD—Severity degree.

**Table 4:** Mean values of storage weight loss (SWL), water activity (A_w_) and lipid oxidation (TBARS) in hamburgers produced from Ross broiler chicken (AP95) without myopathies and affected by wooden breast myopathy after different storage periods.

**SWL (%)**					
A (*n* = 20) ^1^	SD (*n* = 20) ^2^			*p*-value	
	Normal	Moderate	Severe		
30 days	0.35 ± 0.26 ^Cb^	0.73 ± 0.26 ^Cab^	1.10 ± 0.29 ^Ca^	(SD)	<0.0001
60 days	0.91 ± 0.50 ^BCa^	1.72 ± 0.50 ^Ba^	1.57 ± 0.50 ^Ca^	(A)	<0.0001
90 days	1.56 ± 0.37 ^Bb^	1.34 ± 0.38 ^BCb^	12.76 ± 0.36 ^Ba^	(SD × A)	<0.0001
120 days	24.82 ± 0.31 ^Ab^	28.42 ± 0.29 ^Aa^	28.18 ± 0.27 ^Aa^		
**Aw**					
A (*n* = 20) ^1^	Normal	Moderate	Severe	*p*-value	
Start	0.967 ± 0.004 ^Aa^	0.964 ± 0.004 ^Ba^	0.972 ± 0.004 ^Aa^		
30 days	0.908 ± 0.004 ^Ca^	0.896 ± 0.004 ^Db^	0.904 ± 0.004 ^Cab^	(SD)	<0.0001
60 days	0.962 ± 0.006 ^ABa^	0.952 ± 0.006 ^Ca^	0.962 ± 0.004 ^Ba^	(A)	<0.0001
90 days	0.951 ± 0.005 ^Bb^	0.989 ± 0.004 ^Aa^	0.890 ± 0.005 ^Cc^	(SD × A)	<0.0001
120 days	0.878 ± 0.004 ^Dab^	0.872 ± 0.004 ^Eb^	0.886 ± 0.004 ^Ca^		
**TBARS (mg MDA/kg Sample)**					
A (*n* = 20) ^1^	Normal	Moderate	Severe	*p*-value	
Start	2.32 ± 0.12 ^Ca^	1.84 ± 0.12 ^Cb^	1.89 ± 0.12 ^Cb^		
30 days	2.12 ± 0.12 ^Ca^	1.89 ± 0.12 ^Ca^	1.99 ± 0.12 ^Ca^	(SD)	<0.0001
60 days	1.78 ± 0.12 ^Db^	2.21 ± 0.12 ^Ba^	1.97 ± 0.12 ^Cab^	(A)	<0.0001
90 days	4.64 ± 0.16 ^Bb^	5.93 ± 0.13 ^Aa^	3.12 ± 0.12 ^Bc^	(SD × A)	<0.0001
120 days	9.31 ± 0.16 ^Aa^	6.12 ± 0.18 ^Ab^	4.09 ± 0.12 ^Ac^		

^1/A–E^ Means followed by different capital letters in the same column differ from each other by the Tukey test (*p* < 0.05). ^2/a–c^ Means followed by different lowercase letters on the same line differ from each other by the Tukey test (*p* < 0.05). A—Storage, SD—Severity degree.

**Table 5:** Mean values of moisture, fat and protein of hamburgers produced with chicken breast of Ross broiler chickens (AP95) without myopathies and affected by wooden breast myopathy.

Parameters (g/100 g)	Normal	Moderate	Severe	*p*-Value ^1^
Ashes	2.66 ± 0.40	2.46 ± 0.40	2.42 ± 0.40	0.5441
Moisture	72.71 ± 0.28	72.07 ± 0.28	72.80 ± 0.30	0.8786
Fat	4.45 ± 0.21 ^b^	5.32 ± 0.20 ^a^	5.26 ± 0.20 ^a^	<0.0001
Protein	16.16 ± 0.69	17.46 ± 0.69	16.81 ± 0.73	0.0620

^1/a,b^ Means followed by different lowercase letters on the same line are significantly different by the Tukey test (*p* < 0.05).

**Table 6:** Sensory scores (+SEM) of hamburgers produced with chicken breast of Ross broiler chickens (AP95) without myopathies and affected by wooden breast myopathy.

Parameters	Normal	Moderate	Severe	*p*-Value ^1^
Appearance	6.4 ± 0.2 ^b^	6.6 ± 0.1 ^b^	7.0 ± 0.1 ^a^	0.0100
Aroma	7.5 ± 0.1	7.3 ± 0.1	7.3 ± 0.1	0.5021
Flavor	7.7 ± 0.1	7.6 ± 0.1	7.6 ± 0.1	0.8848
Texture	7.0 ± 0.2	7.2 ± 0.2	7.3 ± 0.2	0.2289
Global Acceptance	7.3 ± 0.1	7.3 ± 0.1	7.4 ± 0.1	0.6313

^1/a,b^ Means followed by different lowercase letters on the same line are significantly different by the Tukey test (*p* < 0.05).
